# A Three Species Model to Simulate Application of Hyperbaric Oxygen Therapy to Chronic Wounds

**DOI:** 10.1371/journal.pcbi.1000451

**Published:** 2009-07-31

**Authors:** Jennifer A. Flegg, Donald L. S. McElwain, Helen M. Byrne, Ian W. Turner

**Affiliations:** 1 School of Mathematical Sciences, Queensland University of Technology, Brisbane, Queensland, Australia; 2 Institute of Health and Biomedical Innovation, Queensland University of Technology, Brisbane, Queensland, Australia; 3 School of Mathematical Sciences, University of Nottingham, Nottingham, United Kingdom; University of Oxford, United Kingdom

## Abstract

Chronic wounds are a significant socioeconomic problem for governments worldwide. Approximately 15% of people who suffer from diabetes will experience a lower-limb ulcer at some stage of their lives, and 24% of these wounds will ultimately result in amputation of the lower limb. Hyperbaric Oxygen Therapy (HBOT) has been shown to aid the healing of chronic wounds; however, the causal reasons for the improved healing remain unclear and hence current HBOT protocols remain empirical. Here we develop a three-species mathematical model of wound healing that is used to simulate the application of hyperbaric oxygen therapy in the treatment of wounds. Based on our modelling, we predict that intermittent HBOT will assist chronic wound healing while normobaric oxygen is ineffective in treating such wounds. Furthermore, treatment should continue until healing is complete, and HBOT will not stimulate healing under all circumstances, leading us to conclude that finding the right protocol for an individual patient is crucial if HBOT is to be effective. We provide constraints that depend on the model parameters for the range of HBOT protocols that will stimulate healing. More specifically, we predict that patients with a poor arterial supply of oxygen, high consumption of oxygen by the wound tissue, chronically hypoxic wounds, and/or a dysfunctional endothelial cell response to oxygen are at risk of nonresponsiveness to HBOT. The work of this paper can, in some way, highlight which patients are most likely to respond well to HBOT (for example, those with a good arterial supply), and thus has the potential to assist in improving both the success rate and hence the cost-effectiveness of this therapy.

## Author Summary

In the time it takes you to read this paragraph, one person will have undergone a lower limb amputation due to diabetic foot disease. With the global diabetes population on the rise and set to reach 330 million by 2025, the need for research into therapies and technologies that have the potential to prevent amputation is dire. There is much debate about the best way to treat these wounds, and one treatment that is shrouded with controversy is Hyperbaric Oxygen Therapy (HBOT). There are currently no conclusive data showing that HBOT can assist chronic wound healing, but there has been some clinical success. In light of how expensive properly designed clinical trials can be, we must turn to alternative methods of assessment, such as the theoretical model presented here. The mathematical model reproduces a number of clinical observations. A key result is that while HBOT can assist chronic diabetic wounds, it holds little benefit for wounds that would heal of their own accord. This model represents a useful tool to analyse the optimal protocol, and the results and insights gained from the model may be used to improve both the success rate and thus the cost-effectiveness of this therapy.

## Introduction

Chronic leg ulceration is a significant socioeconomic problem [1]. Those who suffer from leg ulcers experience considerable pain, immobility and decreased quality of life [2]. Approximately 3% of the over 60 age group suffer from lower limb ulceration [3].

A successfully healing wound (or an “acute” wound) is typically thought to progress through four stages; haemostasis, inflammation, proliferation and remodelling [4],[5], although these processes are interconnected and overlapping. Haemostasis should last a matter of hours during which time the blood flow is stopped. Inflammation sees the production of chemoattractants that stimulate fibroblasts, the dominant cell in the proliferative stage of healing, to migrate into the wound site and to produce collagen, the main component of the extracellular matrix (ECM). The cocktail of chemoattractants also stimulate the systematic rearrangement of endothelial cells (ECs) from neighbouring blood vessels [6]. Capillary sprout extension is facilitated by EC proliferation and further migration toward the chemical attractant. The joining of two capillary sprouts within a healing wound forms a loop through which blood can flow and new sprouts develop from this vessel thus propagating angiogenesis [7].

A chronic wound is one in which healing fails to proceed through an orderly and timely process to produce anatomic and functional integrity, or proceeds through the repair process without establishing a sustained anatomic and functional result [8]. The factors responsible for the development of a chronic wound remain unclear, however the most common cause, according to Mathieu [9], is thought to be related to the detrimental effects of prolonged wound hypoxia (oxygen deficiency). Enoch et al. reports that chronic wounds can be arrested in any one of the stages of wound healing, but disruption commonly occurs in the inflammatory or proliferative phases [4].

HBOT involves the intermittent exposure of the body to 100% oxygen at a pressure greater than 1 atmosphere (atm) and its use is supported in the treatment of problem wounds [10]. However, there is much debate about the optimal HBOT protocol in treating such wounds [11]–[14]. Although HBOT is typically used as an adjunctive therapy for treating chronic wounds, many clinicians lack a full knowledge of the evidence-based data that support its use [15].

The primary rationale behind the use of HBOT in the treatment of chronic wounds is to elevate the amount of oxygen delivered to the wound site [16]. For a more detailed review of the wound healing process, the different etiologies of chronic wounds and the use of HBOT to treat nonhealing wounds see Thackham et al. [5]. It should be noted that HBOT is not the only wound healing therapy currently being studied. Gordillo et al. review the use of topical oxygen therapy to assist the closure of chronic wounds [17].

While the wound healing process is undeniably complex, there are useful mathematical models that address various aspects of the phenomenon. The models can be categorised into four broad groups: continuum reaction-diffusion models, mechanochemical models, discrete/stochastic models and multiscale models.

Discrete models have the ability to contain a level of detail that is not possible from a continuum model and, in general, allow for quicker numerical simulation however, continuum models allow mathematical analysis that discrete models do not.

Continuum reaction-diffusion models are arguably the most commonly used theoretical approach for studying the angiogenesis process. One of the first models of this kind is due to Balding and McElwain, who developed a model to investigate tumour-induced angiogenesis [18]. Their model consisted of distinct equations for the blood vessel and capillary tip density. Edelstein had previously used a similar approach to model fungal growth [19]. The concept behind including both blood vessel and tip species is that the ECs in the tip of a vessel guide the ECs in the sprout. This aspect of the model, the so-called “snail-trail” production of blood vessels, means that if the capillary tip density, 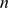, moves with a velocity 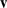, then the rate of increase (that is, production/extension) of blood vessels is given by 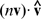, where 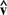 is a unit vector in the direction of 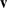. The model by Balding and McElwain accounts for branching and anastomosis but does not account for the extension of vascular loops.

In 1996, Pettet and coworkers developed two models of wound healing angiogenesis [20],[21]. Sherratt, in 2002, described the work in [21] as “the most important theoretical work on wound angiogenesis to date”. In [20] Pettet et al. proposed a three species simplification of the more detailed six species model of wound healing angiogenesis presented in [21] and used an analytic approach to obtain an approximate solution. The first and foremost feature of these models is that they incorporate the dependence of chemoattractant production on the local wound oxygen concentration, with chemoattractant production occurring in a specific oxygen concentration range. Both of these publications modelled the extension of blood vessels using the Balding and McElwain “snail-trail” concept. Other authors have chosen this approach to model wound and tumour angiogenesis (see for example [7],[22],[23]).

In 2002, Gaffney et al. used a two-species model to investigate cutaneous wound healing [24]. Here a travelling wave analysis was used to identify a lower bound on the wave speed of the wound healing unit in terms of two key model parameters, namely, the random motility of capillary tips and the rate of budding of tips. Importantly, Gaffney et al. chose not to use a snail-trail approach, opting rather to consider the EC density explicitly in addition to the capillary tip density [24]. The flux of the capillary tips is determined by random motion and directed motion. The flux of the EC is then assumed to be proportional to the tip flux, where the rate constant is the number of EC that makes up an average capillary tip. More recently, Schugart et al. developed a seven species model of acute wound healing angiogenesis [25], using a similar approach to Gaffney et al. [24] in their treatment of the EC flux, while Addison-Smith et al. used a simple mechanistic model for the sprouting of vessels during tumour-induced angiogenesis [26]. Mantzaris et al. provides an excellent review of continuum models of angiogenesis and concludes that continuum models are important for providing significant insight into the relative importance of different processes [27].

The interactions between cells and the substratum in wound healing are not just chemical; there is also a mechanical influence [27]. For instance, in order to migrate, ECs extend lamellipodia in the direction of migration, and exert tractional forces on the ECM. Mechanochemical models of wound healing are essentially continuum models that account for the forces that cells exert on the ECM. Mechanochemical models of wound healing include the early work by Tranquillo and Murray [28]–[30] and the extensions by Olsen et al. and Cook [31],[32]. These models consider the connection of cells to the ECM and are thus relevant for deeper, dermal wounds. They are typically applied to acute (normal) healing wounds that heal primarily by contraction.

Mechanochemical modelling is needed when considering the remodelling phase of wound healing since it is during this stage that the wound contracts. However, since we are interested in chronic wound healing, and chronic wounds typically arise due to complications in the inflammatory or proliferative phase, the use of mechanochemical models is not addressed here. Furthermore, healing in human wounds is predominantly due to proliferation and migration of cells from outside the wound, whereas in animal models, the contraction of the wound by mechanical forces is thought to be more substantial. It is interesting to note that while the critical role of oxygen in wound healing is well known there are no mechanochemical models, to date, that incorporate angiogenesis.

Discrete models, using cellular automata for example, have been used to capture key features of angiogenesis including the outgrowth, branching and anastomosis of vessels. Stokes and Lauffenburger pioneered the discrete modelling of blood vessel formation in their series of publications [33]–[35]. Their work is discrete in that they present a stochastic model for the random motility and chemotaxis of individual cells. Other aspects of their model use continuum modelling, for example, in the conservation equation for the chemoattractant. Levine and coworkers extended this approach with a series of publications [36]–[40] in which they investigated the mathematical modelling of tumour-induced angiogenesis. In these models, continuous limits of reinforced random walk equations govern the angiogenesis process while ordinary differential equations (ODEs) model the biochemical kinetic equations. In their review, Mantzaris et al. states that one main advantage of using discrete models rather than continuum ones is that individual cells and sprouting of vessels can be tracked [27]. Although discrete models can be computationally fast and efficient, and provide quantitative numerical data, these models are not as readily amenable to mathematical analysis as continuum models are.

Multiscale techniques have been used to simulate the wound healing process (see for example Dallon et al. and Cai et al. [41],[42]). Sun et al. have developed several models of angiogenesis including a multiscale model where the concentration of chemoattractant is modelled at the tissue scale, while the capillary network is modelled at the cellular scale [43],[44]. More recently, Alarcon and coworkers and McDougall and coworkers have used multiscale techniques to investigate angiogenesis associated with tumour growth [45]–[48].

The overall aim of this paper is to use a theoretical model to evaluate the use of hyperbaric oxygen therapy as an adjunct therapy (a therapy used to assist a primary treatment) for treating chronic wounds. Through numerical simulations we conclude that intermittent hyperbaric oxygen therapy has the potential to aid in the healing of chronic wounds (a chronic wound is one which does not heal in an orderly set of stages and in a reasonable amount of time in the way that most wounds do).

## Results

We use the model presented in the Materials and Methods section to simulate a number of different wound healing scenarios, each of which is discussed below.

### Normal Wound without HBOT Exposure

**Figure 1:**
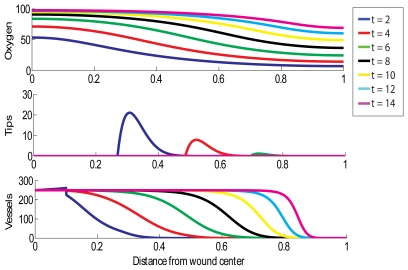
Simulation of successful wound healing. Parameter values for this simulation are shown in Table 1, except here 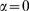. Multiple day intervals are shown (dark blue = 2, red = 4, green = 6, black = 8, yellow = 10, light blue = 12, pink = 14). The top graph shows oxygen tension (mmHg), the middle graph shows tip density (capillaries/cm^2^) and the bottom graph shows blood vessel density (vessels/cm).

We simulate an acute healing wound with the choice of parameters outlined in Table 1, noting that this choice of values yields a steady state oxygen concentration behind the wave front (behind the healing front the oxygen concentration, 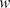, tends to 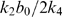 as 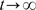) above the lower threshold for capillary tip production, 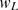, so that healing will be initiated. Fig 1 shows such a normal situation in which a wound of length 2 cm (that is, 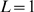) is almost completely reoxygenated within 2 weeks. It would take longer than this, roughly 2.5 weeks, for the simulated wound to completely revascularise. We note that the vessel density can rise above the carrying capacity, 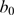, due to rapid chemotaxis and may remain elevated until the remodelling process drives the density to return to normal levels.

**Table 1:** Parameter values.

Parameter	Value	Units	References
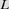	1	cm	Our choice
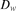	0.18	cm^2^/day	[74],[75]
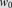	100	mmHg	[76]
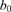	250	vessels/cm	[76]
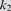	1.04	mmHg/vessel/day	Calculated based on 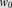 value
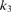	1.3	/day	[35]
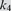	1.3	ml O_2_/ml tissue/day	[78]
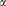	20	dimensionless	[77]
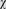	0.01	cm^2^/day/mmHg	Our choice
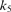	28.8	/cm/day	[80]
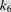	28.8	/cm	[24],[80]
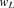	10	mmHg	[81]
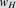	35	mmHg	[81]
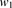	5	mmHg	Our choice
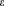	0.1	cm	Our choice

A summary of the parameter values used to simulate successful wound healing.

### Chronic, Stalled Wound without HBOT Exposure

**Figure 2:**
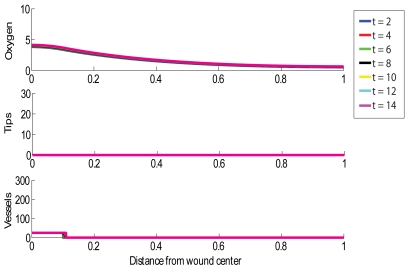
Simulation of a chronic wound in which no healing occurs. Multiple day intervals are shown (dark blue = 2, red = 4, green = 6, black = 8, yellow = 10, light blue = 12, pink = 14). Parameter values: as per Fig 1, except 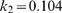.

A chronic wound is simulated by selecting parameter values such that the oxygen concentration behind the injured tissue (near 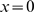 the oxygen concentration tends to 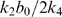) does not rise above the lower threshold for capillary tip production, 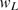. As mentioned above, our assumption is that chronicity is associated with a reduced or impeded supply of oxygen from the vasculature. We therefore reduce the value of 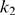 used for the simulation in Fig 1 by a factor of 10 to 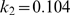. The resulting simulation produces an oxygen profile that is always within the range 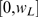 (that is, below the lower threshold for capillary tip production) inside the wound. Fig 2 shows the chronic wound simulation. We note no significant change over time, indicating that no healing is taking place.

### Chronic Wound with HBOT Exposure

**Figure 3:**
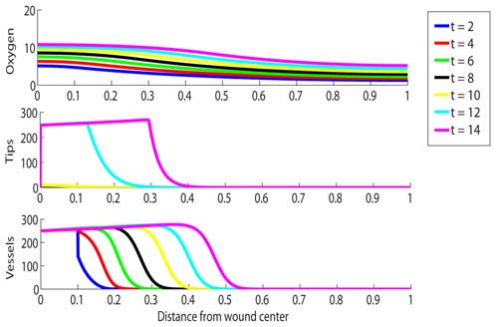
Simulation of the treatment of a chronic wound with HBOT for 1.5 hours per day. Multiple day intervals are shown (dark blue = 2, red = 4, green = 6, black = 8, yellow = 10, light blue = 12, pink = 14). Parameter values: as per Fig 2, except 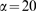.

We now investigate the impact of HBOT on the healing of a chronic wound. The strength and duration of HBOT are given by the parameters 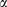 and 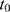 in the model, respectively. The parameter 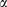 is a measure of the relative increase in supply of oxygen during HBOT compared to times of no treatment. Fig 3 shows such a chronic wound situation under HBOT with 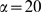 for 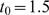 hours per day (that is, 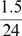 of a day). A value of 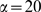 is associated with 100% oxygen at a pressure of just under 3atm (see Materials and Methods Section), which is a reasonable treatment protocol. We note from the simulation that the capillary tip density in the chronic wound reaches highly elevated levels under treament and that healing is quickly initiated in the chronic wound.

### Chronic Wound with Insufficient and Excessive HBOT Exposure

Table 2 shows that an 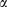 value of 5.73 equates to 100% oxygen at 1atm (that is, normobaric oxygen therapy). The analysis presented later (see Expression (22) in “Analysis of Feasible HBOT Protocol”) predicts that a chronic wound simulated with 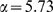 will not heal and this is confirmed by numerical simulations. Thus we have shown, under the assumptions on the model presented here, that normobaric oxygen will not stimulate healing of a chronic wound and we have answered the somewhat controversial question of whether or not normobaric oxygen can be used to substitute for HBOT in the treatment of chronic wounds. Normobaric oxygen fails to stimulate healing in the chronic wound since the oxygen levels under the treatment are still insufficent to initiate capillary tip production. Similarly, our numerical simulations reveal that values of 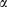 in excess of about 2000 are too high to enable healing to occur. This is because the oxygen levels are raised so much under the treatment that capillary tip production is switched off when the upper oxygen threshold, 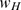, is reached and surpassed. Such high values of 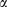 are not considered physically feasible (see Table 2).

**Table 2:** Typical Protocols.

Pressure	Oxygen	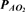	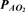	Calculated 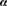
(atm)	level	calculated	clinical	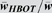	
1	21%	100	89	1	0
1	100%	673	507	6.73	5.73
2	100%	1433	N.A.	14.33	13.33
2.4	100%	1737	N.A.	17.37	16.37
3	100%	2193	1721	21.93	20.93

A summary of values for the parameter 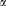 for some typical protocols.

### Chronic Wound with Premature Termination of HBOT

**Figure 4:**
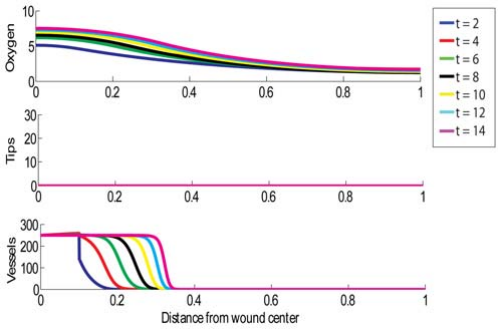
Simulation of the treatment of a chronic wound with HBOT for 1.5 hours per day, where the treatment is stopped after 5 days. Multiple day intervals are shown (dark blue = 2, red = 4, green = 6, black = 8, yellow = 10, light blue = 12, pink = 14). Parameter values: as per Fig 2, except 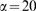.

The results presented in Fig 4 reveal what happens when we simulate a situation in which HBOT is halted prematurely (after 5 days). Interestingly, the effects of HBOT seemed to persist for some time after treatment is halted, but the healing progress slows considerably (compare Figs 3 and 4). Thus, if we want the wound to close as quickly as possible, then HBOT should not be terminated until complete healing of the wound is observed. Note that this is in disagreement with typical clinical protocols, which is to apply the therapy daily for about 6 weeks [16]. This restriction is likely based on cost considerations rather than clinical or experimental evidence which indicates that this is more effective in stimulating healing than continuing until the wound is completely healed.

### Normal Wound with HBOT Exposure

**Figure 5:**
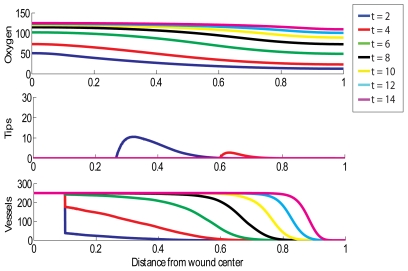
Simulation of a normal wound under HBOT. Multiple day intervals are shown (dark blue = 2, red = 4, green = 6, black = 8, yellow = 10, light blue = 12, pink = 14). Parameter values for this simulation are the same as in Fig 1, except 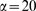.

Many hyperbaric centers around the world advocate the use of HBOT to treat ‘normal’ wounds on the basis that HBOT may accelerate healing in sports injuries [49]. This use of HBOT is highly controversial [50]. Typically a sports injury is internal (muscular) rather than dermal, but here we consider the effect of applying HBOT to a normal healing wound. Comparing Figs 1 and 5, we see that there is little benefit (at most, a 10% increase in the rate at which blood vessels are progressing through the wound space) in applying HBOT to a wound that progresses through the healing process of its own accord. Furthermore, the relatively high-cost of HBOT further detracts from the appeal of its use to treat such wounds. This simulation also shows that the capillary tip density falls significantly towards the end of the healing process. Numerical experimentation reveals that healing will occur, even with very small levels of capillary tips, suggesting that it is the presence of capillary tips, rather than their quantity, that is important for initiating healing. Clinically, this means that stimulating capillary tip production is the crucial factor that enables a chronic wound to heal when HBOT is applied.

We note from Fig 1 and 5 that the density of capillary tips at comparable times is lower in the treated wound than the untreated one. This result can be explained as follows. The normal wound develops a vasculature which supplies sufficient oxygen to the wound to initiate angiogenesis. The wound does not require HBOT to heal. The application of HBOT to this wound increases oxygen levels and reduces the net tip production during times of treatment, resulting in a decreased capillary tip density. In the preceding paragraph, we deemed the therapy to have a positive effect on healing however, this was based on the faster progression of blood vessels through the wound site under the treatment.

### Clinical Implications of Feasible HBOT Protocol Region

We now discuss the potential clinical implications of the restrictions derived for the HBOT protocol, the mathematical detail of which is shown in “Analysis of Feasible HBOT Protocol” in “Materials and Methods”. By considering the feasible HBOT protocol (that is, the range of 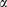 values, where 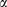 is the relative increase in supply of oxygen during HBOT) to be those that

1. allow the oxygen concentration at the wound edge to rise above 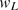 after one session and
2. allow the oxygen concentration at the wound center to fall below 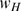 before the start of the next session

where 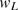 and 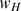 are the lower and upper oxygen concentration thresholds, respectively, for capillary tip production to take place, we are able to derive constraints that depend on key parameter values from the model for the range of feasible HBOT regimes. We note that if a lower bound is too high then a patient would need to be exposed to levels of pressure that are not safe in order for healing to be observed. For instance, if a particular set of wound parameter values lead to a lower bound that exceeds twenty-one, then we must conclude that HBOT will not assist this patient, since only 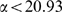 is clinically reasonable (see Table 2).

We consider two approaches to estimating the feasible protocol region. The first approach is to assume that the kinetics dominate the evolution of the oxygen concentration within the wound space and the second is to assume that the blood vessels do not migrate into the wound significantly over the first 24 hours of healing and to solve the resulting partial differential equation (PDE) for the oxygen concentration using Green's functions. We will not consider the upper bound from either approach since both failed to yield clinically relevant restrictions. Instead we focus on the two lower bounds, namely:
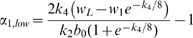
(1a)and
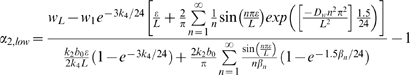
(1b)where 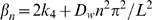 and 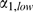 and 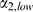 are the lower bounds for the first and second approach, respectively, described above.

We use Eqs (1a) and (1b) to identify patients who are unlikely to benefit from HBOT: these individuals will have higher values of 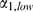 and 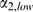. Examining Eqs (1a) and (1b) reveals that patients with the following characteristics are unlikely to benefit from HBOT:

- poor arterial supply of oxygen (reduced 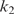). This includes patients that suffer from diseases that lead to vasoconstriction (such as peripheral arterial disease [51] that results in a narrowing of the arteries). Furthermore, patients with sepsis and/or adult respiratory distress syndrome often have abnormalities in oxygen delivery throughout the body [49].
- a high removal of oxygen from the wound tissue (increased 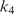). This includes patients with increased levels of bacteria in their wounds, since a high bacterial load is known to increase the consumption of oxygen within the wound space substantially [52].
- excessively hypoxic wounds (reduced 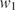). The more hypoxic the wound is when the patient first arrives for treatment, the harder it will be for the wound to reach the conditions needed for capillary tip production and hence successful healing. Chronic wound hypoxia is known to blunt antibacterial activity, collagen synthesis and neovascularization [53].
- dysfunctional EC response to oxygen (higher 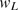). A patient is unlikely to respond to HBOT if their wound inflammatory cells (such as macrophages, fibroblasts and ECs) respond inappropriately to the local level of oxygen. Diseases such as venous hypertension cause veins to become congested with different matrix molecules and locks the wound into a self-amplifying stage of chronic inflammation [54]. In this instance, cells such as fibroblasts loose their ability to respond to chemical stimuli. Similarly, if ECs loose their ability to respond appropriately to oxygen within the wound, then there is a risk of non-responsiveness to HBOT.

## Discussion

In this paper we have developed a simple mathematical model that simulates the healing of both acute and chronic wounds. The modelling framework is based on the premise that chronic wound healing is associated with poor and/or impeded oxygen delivery from the vasculature. We are now in a position to extend the model to investigate other hypotheses. For example, chronic wound healing may be associated with impaired cellular function such as poor cell chemotactic responsiveness [55]. Alternatively, certain chronic wounds may be extremely hypoxic because there is a high bacterial load in the wound bed [56]. Our model can easily be adapted to study this situation by increasing 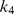, the rate of removal/consumption of oxygen in the equation governing the oxygen distribution, Eq (2a).

The development of new blood vessels occurs by two processes, namely, angiogenesis and vasculogenesis. Here we have only modeled the effect of oxygen on angiogenesis. The extension of the model to include the vasulogenesis and its potential as a mechanism for the improved healing associated with HBOT is a further extension of the model. There has already been some work on the role of vasculogenesis in tumour growth (see for example Stamper et al. [57]).

We used our model to evaluate the effect of treating chronic wounds with HBOT. In summary, our simulations have allowed us to make several, clinically-relevant conclusions including the following:

- intermittent HBOT accelerates the rate of healing of a chronic wound;
- normobaric oxygen therapy can not be used as a substitute for HBOT;
- treatment of a chronic wound with HBOT should not be stopped prematurely but continued until healing is complete. While there is much debate about how many sessions of HBOT should be administered, it has been observed that premature discontinuation of the therapy can have negative or even tragic consequences for the patient [58];
- healing of a chronic wound can occur under HBOT even with very small quantities of capillary tips present, indicating that it is their presence that initiates healing;
- identifying the range of clinically useful HBOT protocols (that is, protocols that stimulate healing) is crucial if HBOT is to enhance the healing process.

By considering the feasible HBOT protocol (that is, the range of 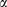 values, where 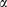 is the relative increase in supply of oxygen during HBOT) to be those that

1. allow the oxygen concentration at the wound edge to rise above 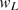 after one session and
2. allow the oxygen concentration at the wound center to fall below 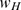 before the start of the next session

where 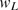 and 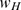 are the lower and upper oxygen concentration thresholds, respectively, for capillary tip production to take place, we were able to derive constraints that depend on key parameter values from the model for the range of feasible HBOT regimes. By considering patients that will need excessive exposure to pressure in order to stimulate healing in conjunction with the lower bounds on the 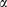 parameter, we predict that patients with any of the following conditions are unlikely to respond well to HBOT:

- poor arterial supply of oxygen,
- high removal of oxygen from the wound tissue,
- excessively hypoxic wounds and
- dysfunctional EC response to oxygen.

In conclusion, we have used a simple three species model of wound healing to evaluate the effect of treating chronic wounds with HBOT. While the causal reasons for the improved healing remain unclear, protocols will remain empirical and an unreliable screening process for appropriate patients will remain in place. The work of this paper is a first step towards identifying in a systematic manner patients who are likely to respond well to HBOT and thus has the potential to assist in improving both the success rate and the cost-effectiveness of this therapy.

## Materials and Methods

We adopt a continuum approach to modelling angiogenesis in wound healing, similar to that of Balding and McElwain [18]. We focus on three key species: oxygen: 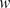, capillary tips: 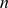 and blood vessels: 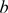. For simplicity, we consider the wound to be one-dimensional, of total length 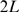 and symmetric about its centre. Here 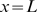 represents the wound centre while 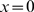 denotes the edge of the wound. Equations governing the evolution of 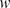, 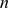 and 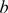 are presented in turn below.

Oxygen concentration, 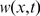:
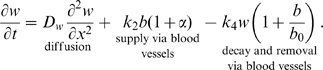
(2a)That diffusion is the primary mechanism by which oxygen is transported through an unvascularised wound site is well established [59] and here we assume a linear diffusion equation for oxygen, with assumed constant diffusivity 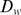. Clearly the formation of new blood vessels to replace the damaged system will increase the supply of oxygen (and other nutrients) to the wound site [51]. We assume that oxygen is supplied by new vessels at rate 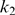. We assume that oxygen is removed via the vasculature at rate 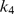. Molecular oxygen is also assumed to decay naturally. HBOT is known to substantially increase oxygen levels within the wound [5],[60] and we thus model the application of HBOT to a wound as an increase in supply during the time of treatment. That is, we take 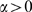 during the session and 0 at all other times.

Capillary Tip density, 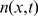:
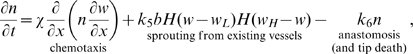
(2b)where 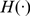 is the Heaviside function. We assume that the dominant mechanism for capillary tip movement is migration down the oxygen gradient (with coefficient 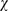). That is, the tips are attracted to regions of relative hypoxia, which is consistent with the experimental literature [61]. We note that, typically chemotaxis is used to model attraction of a species towards a chemical gradient, whereas we are using it here to model the migration of capillary tips down the oxygen gradient and thus the term appears slightly differently than a typical chemotaxis term. Tip sprouting occurs from existing vessels at rate 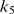 within a range of values of oxygen concentration, 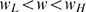. This reflects the well-established concept that proliferation of the ECs that constitute the tips occurs in a region just behind the healing front [62]. Capillary tips are known to be lost due to anastomosis during the healing process [63] and here we model this as a linear process with rate constant 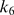.

The idea that the oxygen gradient within the wound tissue is responsible for driving the process of angiogenesis is well-supported in the literature [2], [11], [61], [64]–[66]. For instance, Tompach et al. states that “angiogenesis is driven by a gradient of oxygen whereby high arterial oxygen tension drives angiogenesis into hypoxic spaces” [11] while Oberringer et al. make note that “the oxygen gradient serves as a key stimulus for the direction of cell migration” [61]. While we acknowledge that a gradient of chemoattactant (such as vascular endothelial growth factor (VEGF)) may also be present in the wound, we justify modelling the oxygen concentration as a chemical stimulus without including VEGF by referring to the statement by Bao et al. “a gradient of VEGF expression is established that parallels the hypoxic gradient, and ECs subsequently migrate toward the most hypoxic areas” [67].

There is evidence that oxygen regulates the production of chemoattractant by macrophages and that it is the stimulus provided by the chemoattractants that stimulate angiogenesis [68]–[71]. Pettet et al. avoid the inclusion of a macrophage species in their model by assuming that within a window of oxygen 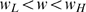, chemoattractants are produced, which in turn stimulate new blood vessel production. Here we avoid the need for explicitly including the chemoattractant by assuming that capillary tips are produced only within the oxygen interval 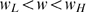. This is consistent with current literature suggesting that in both chronically hypoxic wounds [72] and those that have high levels of oxygen [68], angiogenesis is halted.

Blood vessel density, 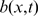:
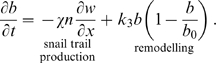
(2c)


We apply the widely used Balding-McElwain “snail trail” approach to modelling the laying down of blood vessels by the moving capillary tips [18],[27]. After the rapid formation of blood vessels during the inflammatory and proliferative stages of healing, the vasculature system is remodelled [73] and we assume that this behaviour can be described by a logistic term with growth rate 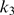 and the normal blood vessel density, 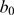, as the “carrying capacity”.

In order to close Eqs (2a)–(2c), we impose boundary and initial conditions as follows. At the wound centre, 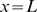, we impose zero flux of oxygen due to the assumed symmetry of the wound about 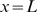, so that:
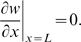
(3a)


We assume that the wound edge is devoid of capillary tips and that the flux of oxygen there is zero so that the wound and tissue oxygen concentrations rapidly equilibrate under intermittent HBOT and we have
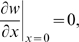
(3b)and
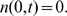
(3c)


We assume that initially the wound is devoid of tips, the blood vessel density is that of normal tissue within a certain area of the wound edge (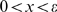) and the wound is partially oxygenated throughout this vascularised region so that
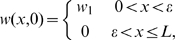
(3d)

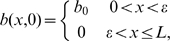
(3e)

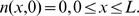
(3f)


For numerical simulations, the discontinuity in the initial condition of the blood vessels is “smoothed” by replacing the two sharp corners with two small quarter-circles.

### Parameter Estimation

#### Wound length, 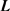


We consider a square wound, with sides of length 2 cm. Due to the assumed symmetry of the wound, this gives 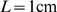.

#### Diffusion Coefficient for Oxygen in Tissue, 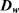


It is generally agreed that the diffusivity of oxygen in water is 2×10^−5^ cm^2^/sec (or, equivalently, 1.728 cm^2^/day). Following Croll et al., the effective diffusivity in a porous medium can be described by Maxwell's solution:
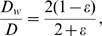
where 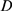 is the oxygen diffusivity in the extracellular fluid (that is, water), 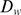 is the effective diffusivity throughout the porous medium and 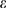 is the volume fraction of intracellular material [74]. Bjornaes et al. claims that the extracellular volume fraction is approximately 0.15 for tumour tissue [75]. Fixing 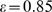 and 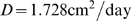 we estimate 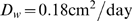.

#### Characteristic oxygen concentration, 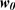


Secomb et al. argues that the average oxygen tension at the entrance of the capillaries is 100 mmHg [76]. This is consistent with estimates from other authors including Piantadosi [77]. We therefore fix 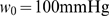.

#### Rate of removal of oxygen from the wound, 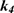


Takahashi et al. calculates an oxygen consumption rate of 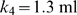 of oxygen per ml of tissue per day which we use in our simulations [78]. We do acknowledge, however, that oxygen consumption rates reported in the literature vary widely.

#### Characteristic vessel density, 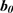


Secomb et al. states that the capillary density is in the range of 4.68–10×10^4^ capillaries/cm^2^ [76]. Assuming that the capillaries are distributed both homogeneously and isotropically, one can assume that, given 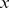 capillaries per cm^2^, a one dimensional cross section of these capillaries will contain approximately 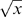 capillaries per cm. This gives the number of vessels per cm to be in the range of 216–316. Accordingly we fix 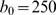.

#### Rate of supply of oxygen to the wound, 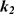


We estimate 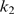 by assuming that the spatially homogeneous steady state for oxygen is identical to the characteristic concentration, 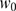. This gives (from Eq (2a)): 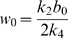, so that 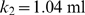 of oxygen per ml of tissue mmHg oxygen/vessel/day.

#### Relative increase in supply of oxygen during HBOT, 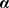


Heyneman et al. states that the oxygen tension in hypoxic wounds is increased from about 10 mmHg to 200 mmHg under HBOT [79]. This indicates that 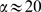 for a standard HBO protocol.

To justify our estimate of the augmented supply of oxygen during HBOT, 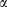, we use a modelling approach that is supported by clinical observations. The (calculated mean) alveolar oxygen tension (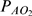) is given by Piantadosi as:
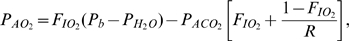
(4)where 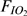 is the inspired oxygen level as a percentage of the total gas, 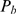 is the barometric pressure (1atm = 760 mmHg), 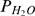 is the water vapour pressure (47 mmHg at 37°C), 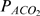 is the mean alveolar carbon dioxide tension (40 mmHg) and 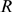 is the respiratory quotient (0.8 when breathing air and 1.0 when breathing 100% oxygen) [66]. Note that Eq (12) is not an empirical formula, rather it is based on the use of an ideal gas law. This formula predicts that if a patient is breathing 21% oxygen at 1atm then the alveolar oxygen tension is 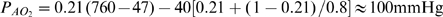. This is taken to be the base level of oxygen in the arteries surrounding the wound in the absence of HBO administration (note that the alveolar and arterial oxygen tensions are comparable [77]). The parameter 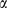 is then assumed to be the ratio of the arterial oxygen tension under HBOT administration to the baseline level.

We now consider the effect that HBOT has on the spatially uniform steady state of oxygen (see Eq (2a)):
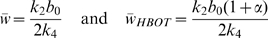
(5)where 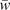 and 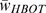 are, respectively, the steady state concentrations in the absence and presence of HBOT, 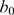 is the blood vessel density steady state, 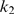 is the rate of supply of oxygen via the vasculature and 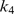 is the rate at which oxygen is removed from the wound site. The relative increase in oxygen tension, predicted by the model, is thus 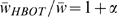, so that the parameter 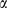 is given by 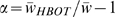. The relative increase in oxygen levels from normal conditions to HBOT is given by ratio of the 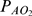 under the HBOT protocol (calculated from Eq (4)) to the 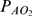 under no HBOT (100 mmHg). Estimates of 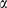 for a variety of protocols are shown in Table 2.

We remark that in existing mathematical models no attempt has been made to base the relative increase of oxygen on either clinical observations or theoretical arguments. For example, Schugart et al. arbitrarily increase the oxygen concentration to 2, 3 and 4 times the normalised levels [25].

#### Rate of sprouting of capillary tips, 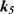


Stokes et al. used data from Sholley et al. to estimate a branching rate of 0.12×10/mm/hour [35],[80]. This corresponds to 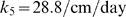.

#### Rate of capillary tip anastomosis, 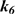


The rate of anastomosis is difficult to estimate. Gaffney et al. argues that it should be of the same order of magnitude as the branching rate so that:
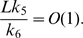
Following Gaffney et al. we therefore take 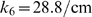 [24].

#### Oxygen window of chemoattractant production, 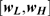


While oxygen is known to regulate the production of angiogenic factors within the wound site [69], there is minimal quantitative data that can be used to estimate the values of 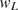 and 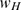. The centre of a normal healing wound is thought to be hypoxic with oxygen tensions of about 25 mmHg [81]. We assume that the window of capillary tip production includes this oxygen concentration. Furthermore, according to Albina et al., maximal hypoxia (of about 10 mmHg) occurs about 4 days post injury [82]. Guided by these data we fix 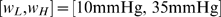.

#### Initial oxygen concentration in vascularised region, 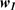


For a chronic wound, we require 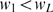 since if 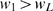 then some tip production occurs and healing is initiated immediately. With 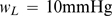, we therefore fix 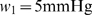.

#### Rate of remodelling of blood vessels, 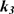


The rate at which the blood vessels are remodelled is also difficult to estimate. Following Gaffney et al., we assume that vessel production is ultimately driven by EC proliferation [24]. The EC proliferation rate was estimated by Stokes et al. to be approximately 0.056/hour [35]. Assuming that vessel production is dominated by EC proliferation and using the above estimate of EC proliferation we arrive at 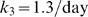. In our simulations, we therefore fix 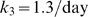, which is in line with Gaffney et al. and Stokes et al. [24],[35].

#### Chemotactic responsiveness of capillary tips to oxygen gradient, 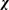


It is difficult to estimate the chemotactic responsiveness of the capillaries to the hypoxic gradient, since, to our knowledge, there is no experimental data available on the migration rate of ECs in various oxygen gradients. Stokes et al. found that the chemotactic responsiveness of EC to a spatial gradient was approximately 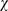 2600 cm^2^/sec/M at a chemical concentration of 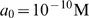 [33]. From this value, we estimate the chemotactic responsiveness of ECs to an oxygen gradient. Typical oxygen concentrations within the wound (that is, 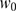) are 100 mmHg. The chemotactic coefficient for fibroblast growth factor (FGF) reported by Stokes et al. is crudely converted to a responsiveness of the EC to an hypoxic gradient by:
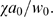
This gives 2.2×10^−4^ cm^2^/day/mmHg. Numerical simulations reveal that a 2 cm wound with this chemotactic responsiveness will take many months to heal. This is not realistic for a normal healing wound, which should proceed through the inflammation and proliferation stages of healing within a few weeks [4],[5]. In fact, Lokmic et al. found that the presence of hypoxia in wound tissue declined after 2 weeks [73]. By using the criterion that the wound should reestablish oxygen levels, roughly speaking, within 2 weeks, we fix 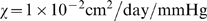.

The parameter values for the normal wound simulation are summarised in Table 1. In a chronic wound, we argue that there is a general decrease in the rate of supply of oxygen due to edema, eschar and bacterial load within the wound [83] and hence that the characteristic oxygen concentration will be significantly reduced. Heyneman et al. states that the oxygen tension in hypoxic wounds is around 10 mmHg (compared to 100 mmHg in normal wounds) [79]. We therefore take this to be the characteristic oxygen tension in a chronic wound. There are many different causes of chronic wounds including diabetes, arterial disease and venous insufficiency. Peripheral neuropathy is considered to be a leading cause in the development of diabetic foot ulcers [84]. This commonly leads to blood flow abnormalities which can divert blood before it has a chance to deliver nutrients and oxygen to the body tissues [85]. We therefore simulate diabetic wound chronicity by reducing 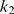, the rate at which the wound is supplied with oxygen, by a factor of 10 (that is, 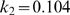 for the chronic simulations). This is the only parameter we change, although we acknowledge that there may be other parameter changes (or combination of changes) that could simulate a stalled, nonhealing wound. The parameters and variables presented here are dimensional, however the parameter 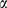 is dimensionless. It is a measure of the relative increase in supply of oxygen during HBOT. Based on both clinical observations and theoretical predictions, 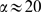 is found to be appropriate for the simulations presented in the Results section.

Advection-dominated initial boundary value problems (IBVPs) arise naturally when developing models of wound healing and in other areas of biology [7],[20],[21],[86],[87]. The numerical method implemented in the case of advection-dominated IBVP must be constructed carefully for reasons outlined previously [88]. The problem presented here is solved using a finite volume method (FVM) with a Roe flux limiter [89], and all simulations are performed with 1500 control volumes.

### Analysis of Feasible HBOT Protocol

We now determine parameter constraints on the HBOT protocol by considering the change in oxygen concentration over the first 24 hours of treatment. Consideration of Eqs (2a)–(2c) reveals that unless the oxygen concentration somewhere within the wound space falls in the range 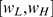, then production of capillary tips is not possible and healing will not occur. We thus define “feasible” 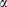 values to be those that

1. allow the oxygen concentration at the wound edge to rise above 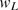 after one session has been completed and
2. allow the oxygen concentration at the wound center to fall below 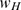 before the start of the next session.

Current clinical protocol is to administer 1.5 hours of treatment once per day. Hence the above two conditions can be expressed mathematically as:

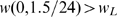
 and

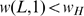
 (Recall that the oxygen concentration, 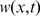, is measured at position, 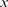, which is given in centimeters and time, 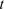, which is given in days.)

We consider two approaches to deriving the aforementioned constraints. The first involves assuming that the kinetics of Eq (2a) dominate the oxygen concentration within the wound space. This allows us to consider a time-dependent ODE for the oxygen concentration which is solved using standard techniques. The second approach is to assume that the blood vessels do not migrate into the wound significantly over the first 24 hours of healing. The resulting partial differential equation (PDE) for the oxygen concentration decouples from the remaining equations and can be solved using Green's functions [90].

By assuming that the kinetics dominate the evolution of oxygen within the wound space we arrive at the following ODE that governs the transition from the steady state oxygen concentration without HBOT to the steady state concentration under treatment
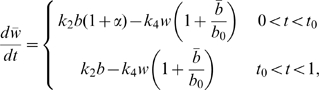
(6)where 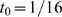, represents the proportion of each day that a patient is administered HBOT (that is, 90 minutes per day). From Eq (2a), the blood vessel density at steady state is 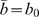. Substituting this into Eq (6) we obtain
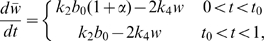
(7)with 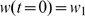.

Eq (7) has solution
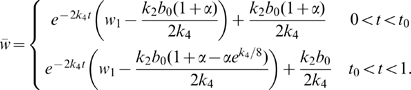
(8)


Using Eq (8) it is straight forward to show that our conditions for the feasible HBOT protocol as outlined previously can be written:

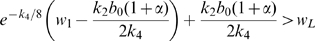
,

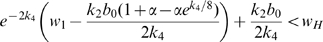
.

These inequalities identify a region of 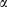 values (that is, HBOT protocols) for treating a chronic wound such that pro-healing effects are predicted (under the given assumptions) to be observed:
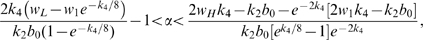
(9)where capillary tip production can only take place in the oxygen range of 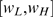, 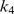 is the rate at which oxygen is removed from the wound via the vasculature, 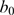 is the characteristic blood vessel density, 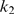 is the rate at which oxygen is supplied by the blood vessels and 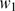 is the initial oxygen concentration at the wound edge.

It should be noted that this analysis is based on the assumption that the wound is chronic in that, if it is left untreated, then the steady state oxygen concentration will not rise above the lower threshold for capillary tip production (that is, 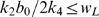).

By substituting the estimated parameter values shown in Table 1 into the inequality in (9), we predict that for this particular set of parameter values, HBOT will assist healing if:
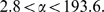
Recall that the parameter 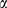 is dimensionless. It represents the increase in supply of oxygen during HBOT relative to periods without the treatment. It should also be noted that the upper limit of this region of feasible values is outside what would be considered “clinically reasonable”.

Numerical experimentation reveals that a chronic wound exposed to HBOT with 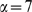 will not heal, which is in violation of the inequalities predicted by the above analysis. There are a number of potential reasons why our lower bound does not provide an accurate restriction on the HBOT protocol including:

1. the oxygen kinetics are not dominant and
2. the blood vessel density does not reach steady state levels during the first day of treatment.

**Figure 6:**
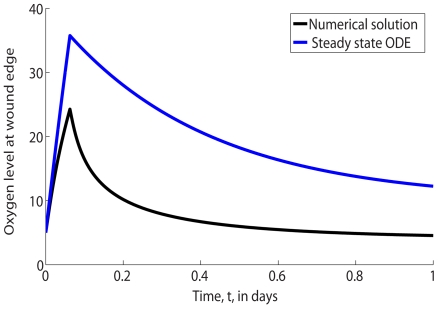
Comparison of the full numerical solution (shown in black) to Eqs (2) and steady state analytic oxygen concentration (shown in blue) at the edge of the wound, 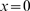. Parameter values: as per Table 1 except 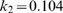.

To emphasize the fact that the steady state approach is inappropriate for deriving the constraints on the feasible HBOT regime, let us consider a chronic wound exposed to HBOT with 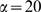. Fig 6 compares the oxygen concentration at the wound margin (that is, at 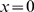) during the first day of healing with the value predicted from the above steady state analysis. Note from Fig 6 that the concentration of oxygen at the wound edge differs substantially from the value associated with the steady state analysis.

On closer inspection Fig 3 reveals that during the first day, the blood vessels do not migrate deep into the wound. By assuming that the blood vessel distribution throughout the wound domain does not change from the initial conditions during the first day of treatment then the oxygen PDE decouples and we have:
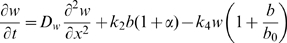
(10)where 
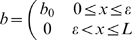
 and subject to the boundary conditions 
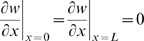
. We find the solution to Eq (10) over the first day of treatment using Green's functions:
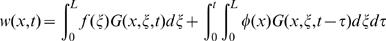
where 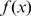 is the initial distribution of oxygen (see Eq (3d)), 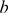 is given above, 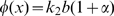 and
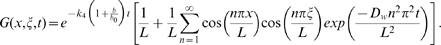
Evaluating the integrals gives:
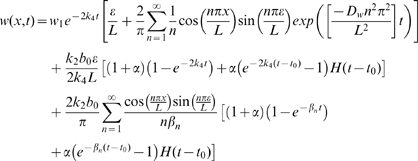
(11)where 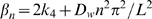, 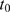 is the duration of the HBOT session on the first day of treatment and 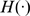 is the Heaviside function.

By imposing 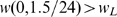 we find:
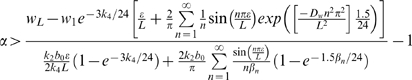
(12)and similarly, we can ensure that 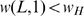 if 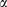 does not exceed:
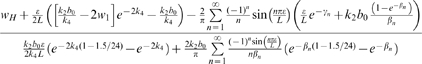
(13)where 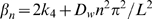 and 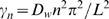. In terms of the estimated parameter values, shown in Table 1, the region of feasible 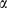 predicted by using Green's function is given by:
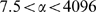
(14)


This lower bound is consistent with numerical simulations, which reveal that healing does not occur with 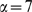. However, we note that the upper limit is significantly higher than what would be considered clinically reasonable.

**Figure 7:**
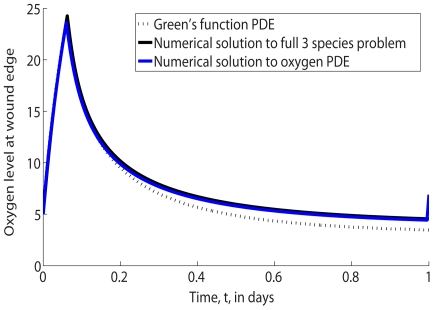
Comparison of the full numerical solution (shown in black) to Eqs (2), the numerical solution to the subproblem (shown in blue) in Eq (10) and Green's function analytic oxygen concentration (see Eq (11)) at the edge of the wound (shown in dashed black), 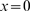. Parameter values: as per Fig 6.

In Fig 7 we compare the oxygen concentration at the wound margin during the first day of healing with that predicted using the Green's function analysis. We note that there is excellent agreement between the values obtained by solving the full model numerically and by using the Green's function approach.

It should be noted that in practice only the lower bounds presented here are useful since exposing a patient to high levels of oxygen for even short periods of time causes oxygen toxicity [91]. In fact, 100% oxygen for 3 hours at 3 atm can cause central nervous system breakdown [50]. Hence a value of 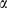 greater than 20.93 (see Table 2) should be considered clinically irrelevant.
